# Application of Autologous Platelet-Rich Plasma on Tooth Extraction Site Prevents Occurence of Medication-Related Osteonecrosis of the Jaws in Rats

**DOI:** 10.1038/s41598-018-37063-y

**Published:** 2019-01-10

**Authors:** Luan Felipe Toro, João Martins de Mello-Neto, Fernanda Furuse Ventura dos Santos, Letícia Chaves Ferreira, Cristian Statkievicz, Luciano Tavares Ângelo Cintra, João Paulo Mardegan Issa, Rita Cássia Menegati Dornelles, Juliano Milanezi de Almeida, Maria José Hitomi Nagata, Valdir Gouveia Garcia, Leticia Helena Theodoro, Cláudio Aparecido Casatti, Edilson Ervolino

**Affiliations:** 1 São Paulo State University (UNESP), School of Dentistry, Department of Basic Sciences, Rua José Bonifácio, 1193, CEP, 16015-050 Araçatuba, SP Brazil; 2 São Paulo State University (UNESP), Institute of Biosciences, Rua Prof. Dr. Antônio Celso Wagner Zanin, 250, CEP, 18618-689 Botucatu, SP Brazil; 3 São Paulo State University (UNESP), School of Dentistry, Department of Surgery and Integrated Clinic, Rua José Bonifácio, 1193, CEP, 16015-050 Araçatuba, SP Brazil; 4 São Paulo State University (UNESP), School of Dentistry, Department of Restorative Dentistry, Rua José Bonifácio, 1193, CEP, 16015-050 Araçatuba, SP Brazil; 5 São Paulo University (USP), School of Dentistry, Department of Morphology, Physiology and Basic Pathology, Avenida do Café, s/n, CEP, 14040-904 Ribeirão Preto, SP Brazil

## Abstract

This study evaluated the effects of local application of autologous platelet-rich plasma (PRP) on the tooth extraction site of rats presenting the main risk factors for medication-related osteonecrosis of the jaw (MRONJ). For seven weeks, senile rats were submitted to systemic treatment with vehicle (VEH and VEH-PRP) or 100 μg/Kg of zoledronate (ZOL and ZOL-PRP) every three days. After three weeks, the first lower molar was extracted. VEH-PRP and ZOL-PRP received PRP at the tooth extraction site. Euthanasia was performed at 28 days postoperatively. Clinical, histopathological, histometric and immunohistochemical analyses were carried out in histological sections from the tooth extraction site. ZOL showed lower percentage of newly formed bone tissue (NFBT), higher percentage of non-vital bone tissue (NVBT), as well as higher immunolabeling for TNFα and IL-1β. In addition, ZOL presented lower immunolabeling for PCNA, VEGF, BMP2/4, OCN and TRAP. VEH and ZOL-PRP showed improvement in the tooth extraction site wound healing and comparable percentage of NFBT, VEGF, BMP2/4 and OCN. Local application of autologous PRP proved a viable preventive therapy, which is safe and effective to restore tissue repair capacity of the tooth extraction site and prevent the occurrence of MRONJ following tooth extraction.

## Introduction

Bisphosphonates (BPs) are very effective antiresorptive drugs^1,2^ for treatment of osteoporosis, Paget’s disease, osteogenesis imperfecta, multiple myeloma, as well as bone pain control, hypercalcemia modulation and inhibition of bone metastasis progression in osteotropic malignant tumors^3–5^. The medication-related osteonecrosis of the jaw (MRONJ) stands out among the adverse events caused by the use of BPs. The American Association of Oral and Maxillofacial Surgery (AAOMS) defines MRONJ as presence of exposed bone in the maxillofacial region for a period longer than eight weeks, in patients submitted to previous or current treatment with antiresorptive drugs and no prior history of radiotherapy on the jaws^6^.

The incidence of MRONJ is 1:10.000–1:100.000 in patients making oral use of BPs in osteoporotic dosage. However, this incidence significantly increases to 1:10–1:100 patients, when BPs are intravenously administered in oncologic doses^7^. Most cases of MRONJ are associated with intravenously administered nitrogen-containing BPs, among which zoledronate stands out as the most potent one. Patients most affected by this condition are women at an advanced age undergoing adjuvant therapy with BPs for cancer treatment, predominantly of multiple myeloma or breast cancer. The jaw is the most affected bone and the two main local risk factors are tooth extraction and periodontal disease^8,9^.

MRONJ was first described by Marx^10^ and its pathogenesis has not yet been fully elucidated. Several possible etiopathogenic factors of MRONJ have been raised concerning the use of BPs: i) induction of severe inhibition of osteoclast activity, preventing bone remodeling and resulting in non-vital bone accumulation; ii) toxic action on oral mucosa cells, reducing soft tissue repair ability, consequently impairing the underlying bone tissue; iii) antiangiogenic effect, resulting in both impairment of tissue repair and avascular necrosis of bone tissue; iv) increased infection susceptibility, since the drug facilitates adherence and bacteria colonization in the exposed bone in the oral cavity; v) dysfunction of the local immune response, resulting in impairment of the mechanisms of both defense and tissue repair^11–14^.

The limited understanding of the pathogenesis of MRONJ greatly restricts its prevention and treatment, and a standard protocol has not been established so far. Antibiotic therapy has been predominantly used as prevention^15^; however, it may not always work efficiently^16^. MRONJ treatment has been carried out by different clinical approaches, based on the clinical staging of the disease. Therapy has been drug-based and/or surgical. Drug therapy consists mostly of the extended use of antimicrobial agents^17–19^. Surgical therapy ranges from conservative to aggressive, by means of curettage and/or sequestrectomy to resection of the jaws^17–19^. MRONJ treatment is time-consuming and generally results in failure or severe side effects^17–19^.

The biological properties of autologous platelet-rich plasma (PRP) make it a potential preventive therapy for MRONJ. PRP consists of a high concentration of platelets in a small volume of blood plasma^20,21^. Activated platelets, in a fibrin matrix, are a source of bioactive molecules that promote activation, proliferation and differentiation of a variety of cell types^22^. Studies show that PRP is able to accelerate the repair process of both bone tissue^23–26^ and soft tissues, including epithelial and connective tissues^27–30^. Moreover, it has been reported that the use of PRP has an anti-inflammatory action^31^ and antimicrobial effect^32^. Although the use of surgical debridement in combination with autologous PRP application for treating severe cases of MRONJ has been successful^33^, its effectiveness as a preventive therapy for this condition has not yet been properly evaluated. Therefore, the aim of this study was to evaluate the effects of local application of autologous PRP in the tooth extraction site in rats exhibiting the main risk factors for MRONJ.

## Results

### General health status, intra-oral and tooth extraction site clinical features

The general health conditions of animals used in this study remained constant and there was no loss of animals throughout the experimental period. The animals tolerated well the drug treatment, blood collection via cardiac puncture and first molar extraction. At the end of the experimental period, there was no statistically significant difference in the mean body weight of animals.

The intra-oral examination showed no macroscopic lesions in the oral cavity and tooth extraction site in groups VEH, VEH-PRP and ZOL-PRP. In contrast, great area of bone exposure (more than half of extraction socket) and impairment of mucous membrane repair were observed in the extraction site in four rats from ZOL group. The remaining rats exhibited similar clinical features; however, with small area of exposed bone (less than half of extraction socket). The scores, distribution of specimens, medians and interquartile ranges of attributed scores according to the clinical aspect of the tooth extraction site in VEH, VEH-PRP, ZOL and ZOL-PRP groups at 28 days postoperatively are presented in Table 1.

**Table 1:** Scores, distribution of specimens, medians and interquartile ranges of attributed scores according to clinical analysis of tooth extraction site and adjacent tissues in VEH, VEH-PRP, ZOL and ZOL-PRP groups at 28 postoperative days.

Clinical Analysis				
Parameter and Respective Scores	Number of Specimens			
	Experimental Groups			
	VEH (n = 7)	VEH-PRP (n = 7)	ZOL (n = 7)	ZOL-PRP (n = 7)
**Clinical Aspect of Tooth Extraction Site and Adjacent Tissues**				
(1) absence of exposed bone and totally repaired mucous membrane	7	7	—	3
(2) absence of exposed bone and partially repaired mucous membrane	—	—	—	4
(3) large extraction site with small area of exposed bone (less than half of extraction socket) and impairment of mucous membrane repair	—	—	3	—
(4) large extraction site with great area of exposed bone (more than half of extraction socket) and impairment of mucous membrane repair	—	—	4	—
medians and interquatile ranges	1 (1–1)	1 (1–1)	4^†,‡^ (3–4)	2^¶^ (1–2)

Symbols: ^†^Statistically significant difference in relation to VEH; ^‡^Statistically significant difference in relation to VEH-PRP; ^¶^Statistically significant difference in relation to ZOL.

### Qualitative and quantitative aspects of PRP samples

PRP smear samples showed structural integrity of platelets. The PRP samples from VEH-PRP and ZOL-PRP groups had at least 3.5 times more platelets than the blood samples that originated them.

### Histopathological aspect of tooth extraction site and adjacent tissues

Parameters, scores, distribution of specimens, medians and interquartile ranges of attributed scores according to histopathological analysis at 28 days after tooth extraction in VEH, VEH-PRP, ZOL and ZOL-PRP are shown in Table 2.

**Table 2:** Parameters, scores, distribution of specimens, medians and interquartile ranges of attributed scores according to histopathological analysis at 28 postoperative days after tooth extraction in VEH, VEH-PRP, ZOL and ZOL-PRP groups.

Histopathological Analysis				
Parameters and Respective Scores	Number of Specimens			
	Experimental Groups			
	VEH (n = 7)	VEH-PRP (n = 7)	ZOL (n = 7)	ZOL-PRP (n = 7)
**Intensity of Local Inflammatory Response**				
(1) absence of inflammation	7	7	—	4
(2) small quantity of inflammatory cells	—	—	—	3
(3) moderate quantity of inflammatory cells	—	—	3	—
(4) large quantity of inflammatory cells	—	—	4	—
medians and interquatile ranges	1 (1–1)	1 (1–1)	4^†,‡^ (3–4)	1^¶^ (1–2)
**Inflammation Extension**				
(1) absence of inflammation	7	7	—	4
(2) partial extension of connective tissue	—	—	—	3
(3) entire extension of connective tissue, without reaching bone tissue	—	—	2	—
(4) entire extension of connective tissue and bone tissue	—	—	5	—
medians and interquatile ranges	1 (1–1)	1 (1–1)	4^†,‡^ (3–4)	1^¶^ (1–2)
**Cellular Pattern and Epithelial Tissue Structure**				
(1) moderate layer of epithelial tissue completely recovering extraction site	4	5	—	3
(2) thin layer of epithelial tissue completely recovering extraction site	3	2	—	4
(3) thin layer of epithelial tissue only in edges of open surgical wound	—	—	4	—
(4) absence of epithelial tissue on open surgical wound	—	—	3	—
medians and interquatile ranges	1 (1–2)	1 (1–2)	3^†,‡^ (3–4)	2^¶^ (1–2)
**Cellular Pattern and Connective Tissue Structure**				
(1) moderate quantity of fibroblasts and large quantity of collagen fibers	4	5	—	3
(2) moderate quantity of both fibroblasts and collagen fibers	3	2	—	4
(3) small quantity of both fibroblasts and collagen fibers	—	—	2	—
(4) severe tissue disorganization with necrosis areas	—	—	5	—
medians and interquatile ranges	1 (1–2)	1 (1–2)	4^†,‡^ (3–4)	2^¶^ (1–2)
**Cellular Pattern and Bone Tissue Structure**				
(1) absence of non-vital bone in tissues adjacent to the extraction site and trabecular bone filling more than half of extraction socket	6	7	—	4
(2) absence of non-vital bone in tissues adjacent to the extraction site and trabecular bone filling less than half of extraction socket	1	—	—	2
(3) presence of few areas with non-vital bone in tissues adjacent to the extraction site and trabecular bone filling less than a third of extraction socket	—	—	2	1
(4) presence of many areas with non-vital bone in tissues adjacent to the extraction site and trabecular bone filling less than a third of extraction socket	—	—	5	—
medians and interquatile ranges	1 (1–2)	1 (1–1)	4^†,‡^ (3–4)	1^¶^ (1–3)
**Contamination Pattern of Tooth Extraction Site**				
(1) presence of bacteria diffusely distributed in extraction site, typical of a normal condition	7	7	—	4
(2) presence of large colonies of bacteria in soft tissues over extraction socket	—	—	—	2
(3) presence of large colonies of bacteria in surface of alveolar bone and in the interior of the extraction socket	—	—	—	1
(4) presence of large colonies of bacteria involving necrosed bone and/or in medullar spaces and in tissues adjacent to the extraction socket	—	—	7	—
medians and interquatile ranges	1 (1–1)	1 (1–1)	4^†,‡^ (4–4)	1^¶^ (1–3)

Symbols: ^†^Statistically significant difference in relation to VEH; ^‡^Statistically significant difference in relation to VEH-PRP; ^¶^Statistically significant difference in relation to ZOL.

**Figure 1:**
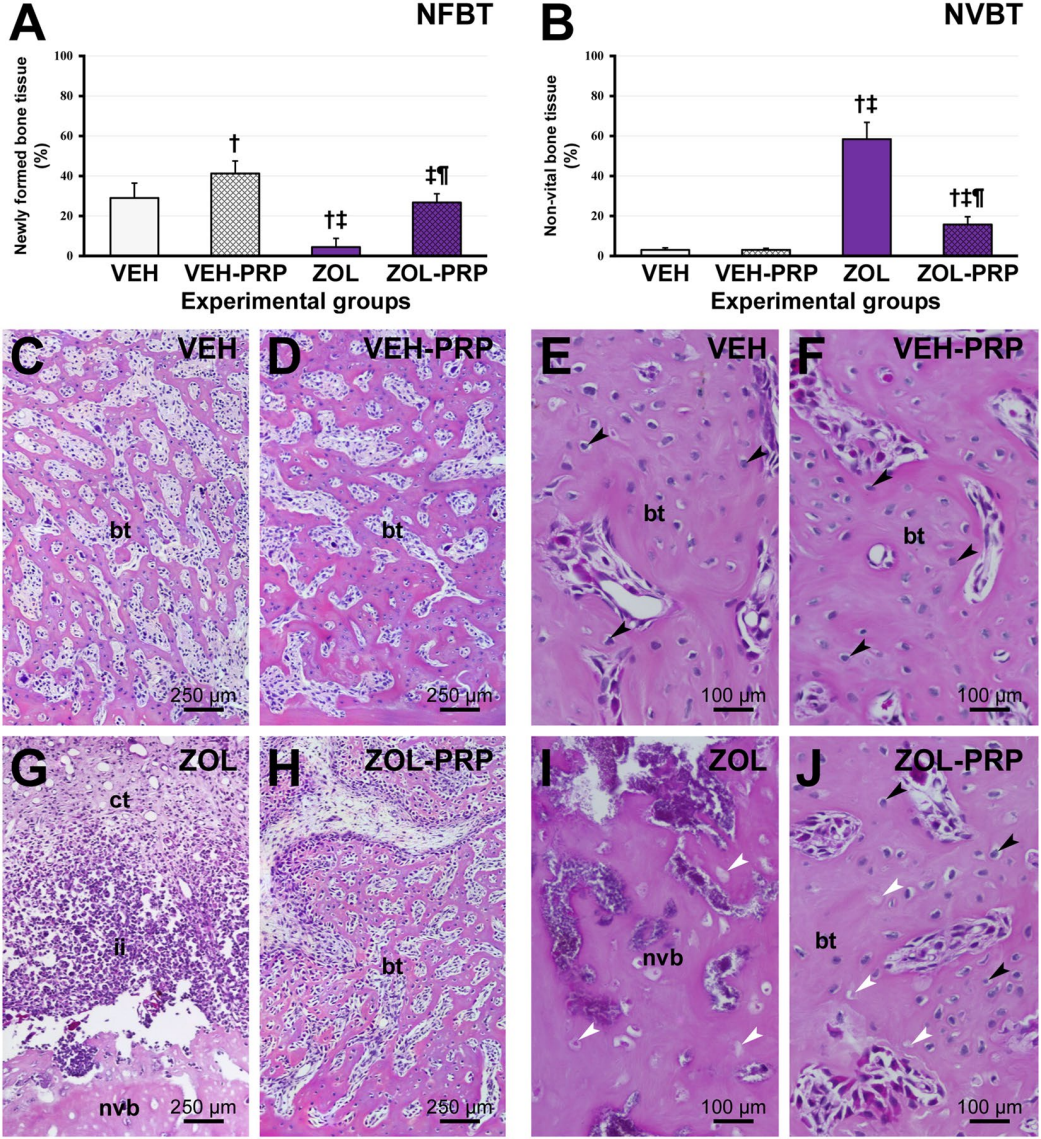
Newly formed bone tissue (NFBT) in tooth extraction site and non-vital bone tissue (NVBT) in tissues adjacent to tooth extraction site. (**A**,**B**) percentage of NFBT (**A**) and NVBT (**B**) in the different experimental groups at 28 postoperative days. (**C–J**) Photomicrographs showing percentage of NFBT in extraction site previously occupied by mesial root of first molar in groups (**C**,**D**,**G**,**H**) and NVBT in areas adjacent to extraction site (**E**,**F**,**I**,**J**) in VEH (**C**,**E**), VEH-PRP (**D**,**F**), ZOL (**G**,**I**) and ZOL-PRP (**H**,**J**). Symbols: bt, bone tissue; black arrows, osteocytes; ct, connective tissue; ii, inflammatory infiltrate; nvb, non-vital bone; white arrows, empty lacunae or occupied by necrotic remains of osteocytes; ^†^Statistically significant difference in relation to VEH; ^‡^Statistically significant difference in relation to VEH-PRP; ^¶^Statistically significant difference in relation to ZOL. Staining: HE. Original magnification: (**C**,**D**,**G**,**H**) 100×; (**E**,**F**,**I**,**J**) 250×. Scale bars: (**C**,**D**,**G**,**H**) 250 μm; (**E**,**F**,**I**,**J**) 100 μm.

In group ZOL, absence of epithelial tissue regeneration in the mucosa overlying the tooth extraction site and persistent inflammation in the lamina propria was observed. The interior of the extraction socket was empty or filled with small amount of trabecular bone and moderate amount of connective tissue. When present, bone tissue in the extraction socket was comprised of thin trabecular bone and large medullary spaces. The connective tissue at this site was composed of a large amount of inflammatory cells, a moderate amount of both fibroblasts and blood vessels, as well as a small amount of collagen fibers. Numerous and extensive areas of non-vital bone tissue interspersed with vital bone tissue were found surrounding the extraction socket. In this group, all specimens had non-vital bone areas involved with significant inflammatory cells and circumscribed by microbial colonies (Fig. 1).

VEH, VEH-PRP and ZOL-PRP groups showed similar histopathological characteristics. The extraction sockets were covered by epithelial tissue associated with connective tissue rich in fibroblasts, collagen fibers, blood vessels and some inflammatory cells. The interior of the extraction sockets were, in most specimens, predominantly filled by bone tissue, composed of moderately thick trabecular bone involving small medullary spaces filled with loose connective tissue and/or bone marrow. Large areas of non-vital bone tissue were absent in VEH and VEH-PRP. In group ZOL-PRP, larger amounts of empty osteocyte lacunae and larger amounts of small areas of non-vital bone tissue interspersed with vital bone tissue were clearly observed. In these groups, bacterial colonies were not observed in the bone tissue (Fig. 1).

### NFBT and NVBT in tooth extraction site and adjacent tissues

NFBT in the tooth extraction site was significantly lower in ZOL group, than in the other groups. There was no statistically significant difference between VEH and ZOL-PRP. NFBT was significantly higher in VEH-PRP than in VEH and ZOL-PRP (Fig. 1).

The NVBT in ZOL and ZOL-PRP was significantly higher than in VEH and VEH-PRP groups. NVBT was significantly lower in ZOL-PRP than in ZOL group. No statistically significant difference was observed between VEH and VEH-PRP groups (Fig. 1).

### Immunolabeling pattern for TNFα, IL-1β, PCNA, VEGF, BMP2/4, OCN and TRAP

**Figure 2:**
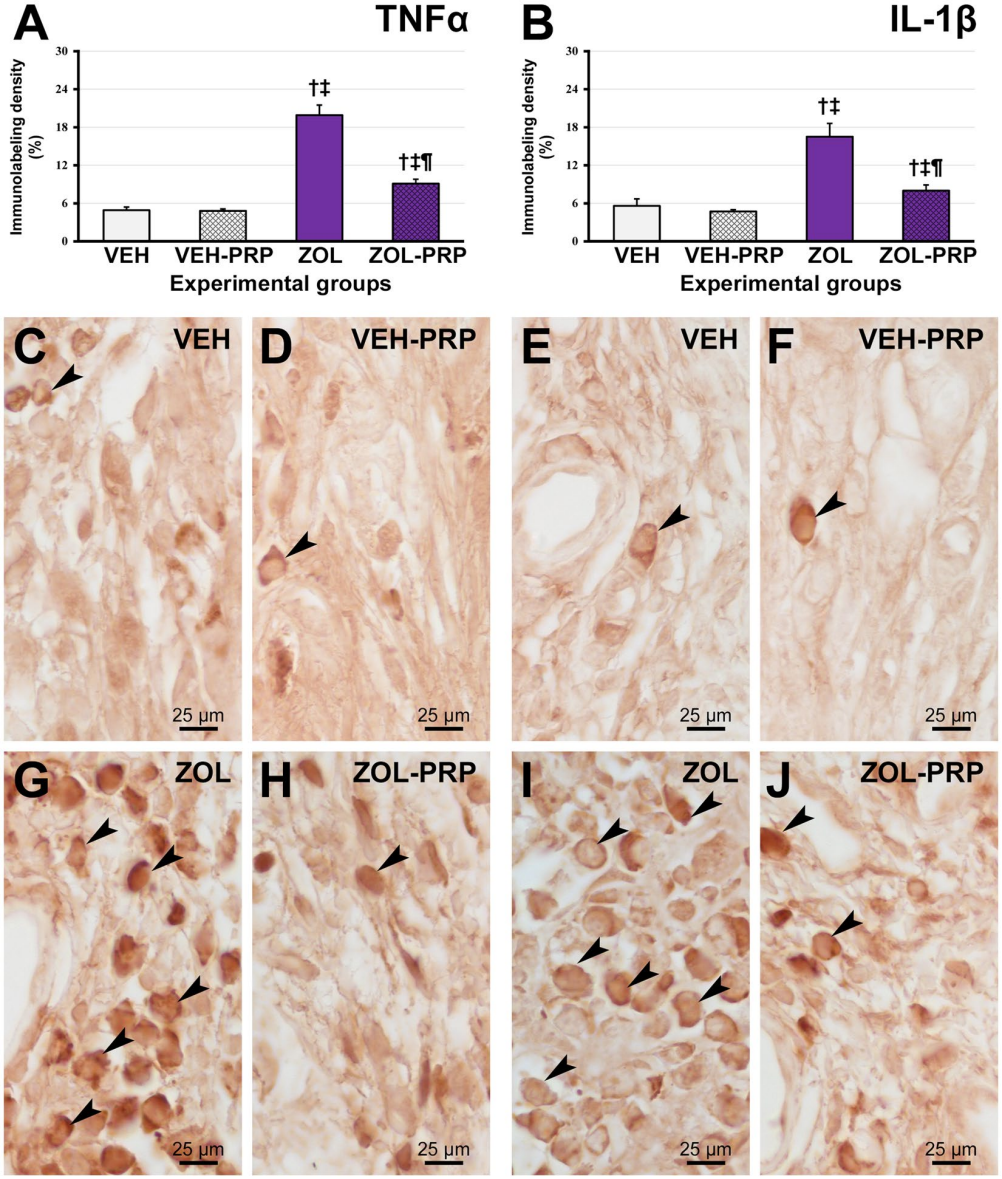
Immunolabeling pattern for TNFα and IL-1β in tooth extraction site. (**A**,**B**) Graphic presenting immunolabeling data for TNFα (**A**) and IL-1β (**B**) in tissue of extraction site in the different experimental groups at 28 postoperative days. (**C**–**J**) Photomicrographs showing immunolabeling pattern for TNFα (**C**,**D**,**G**,**H**) and IL-1β (**E**,**F**,**I**,**J**) in VEH (**C**,**E**), VEH-PRP (**D**,**F**), ZOL (**G**,**I**) and ZOL-PRP (**H**,**J**). Symbols: arrows, immunolabeling cells; ^†^statistically significant difference in relation to VEH; ^‡^Statistically significant difference in relation to VEH-PRP; ^¶^Statistically significant difference in relation to ZOL. Original magnification: 1000×. Scale bars: 25 μm.

**Figure 3:**
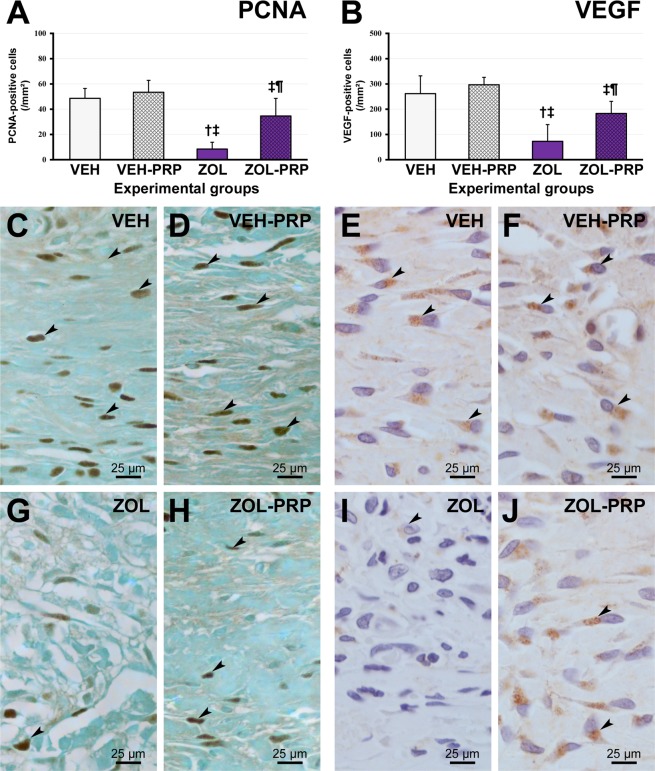
Immunolabeling pattern for PCNA and VEGF in tooth extraction site. (**A**,**B**) Graphic presenting immunolabeling data for PCNA (**A**) and VEGF (**B**) in tissue of extraction site in the different experimental groups at 28 postoperative days. (**C**–**J**) Photomicrographs showing immunolabeling pattern for PCNA (**C**,**D**,**G**,**H**) and VEGF (**E**,**F**,**I**,**J**) in VEH (**C**,**E**), VEH-PRP (**D**,**F**), ZOL (**G**,**I**) and ZOL-PRP (**H**,**J**). Symbols: arrows, immunolabeling cells; ^†^Statistically significant difference in relation to VEH; ^‡^Statistically significant difference in relation to VEH-PRP; ^¶^Statistically significant difference in relation to ZOL. Original magnification: 1000×. Scale bars: 25 μm.

**Figure 4:**
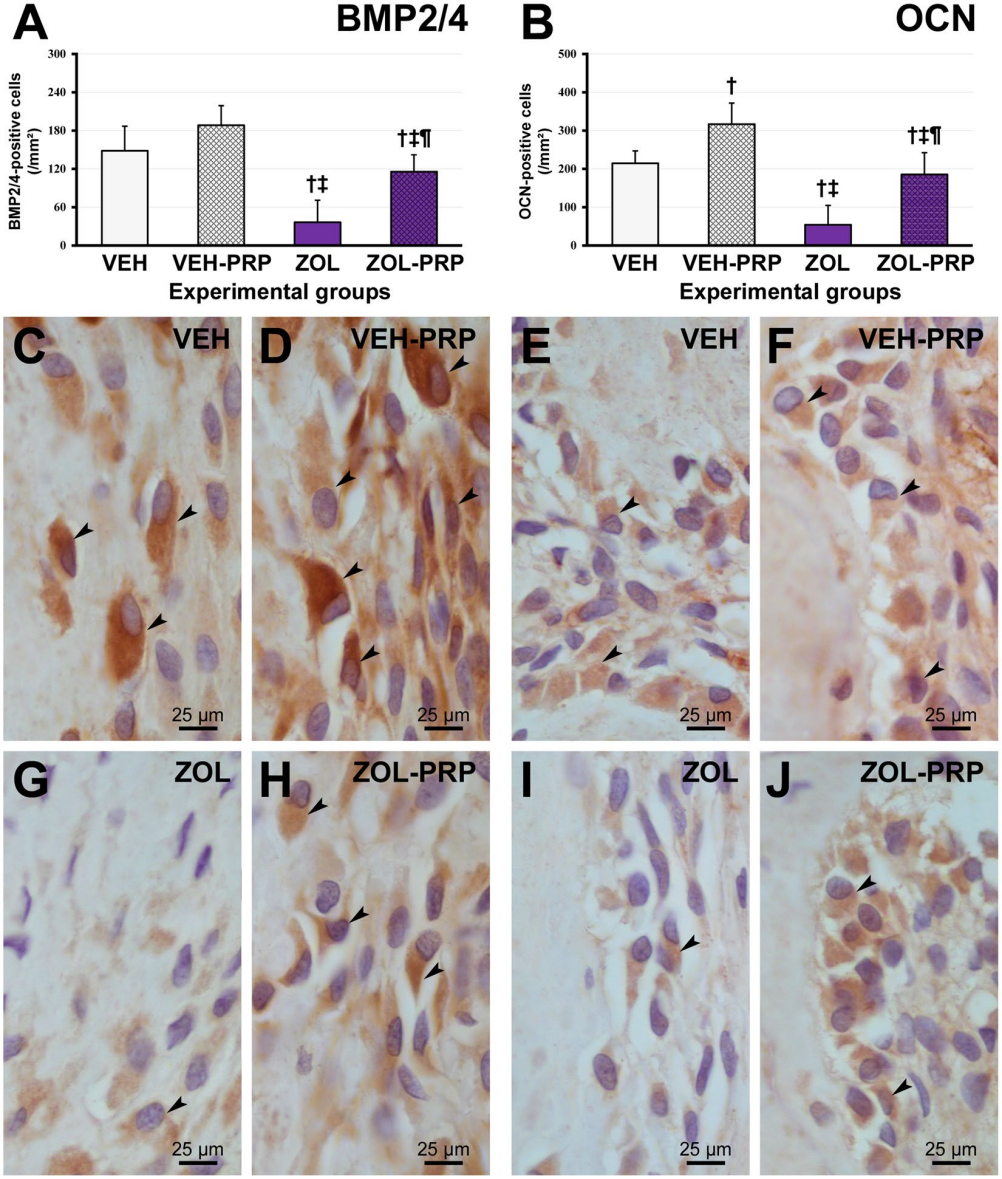
Immunolabeling pattern for BMP2/4 and OCN in tooth extraction site. (**A**,**B**) Graphic presenting immunolabeling data for BMP2/4 (**A**) and OCN (**B**) in tissue of extraction site in the different experimental groups at 28 postoperative days. (**C**–**J**) Photomicrographs showing immunolabeling pattern for BMP2/4 (**C**,**D**,**G**,**H**) and OCN (**E**,**F**,**I**,**J**) in VEH (**C**,**E**), VEH-PRP (**D**,**F**), ZOL (**G**,**I**) and ZOL-PRP (**H**,**J**). Symbols: arrows, immunolabeling cells; ^†^Statistically significant difference in relation to VEH; ^‡^Statistically significant difference in relation to VEH-PRP; ^¶^Statistically significant difference in relation to ZOL. Original magnification: 1000×. Scale bars: 25 μm.

**Figure 5:**
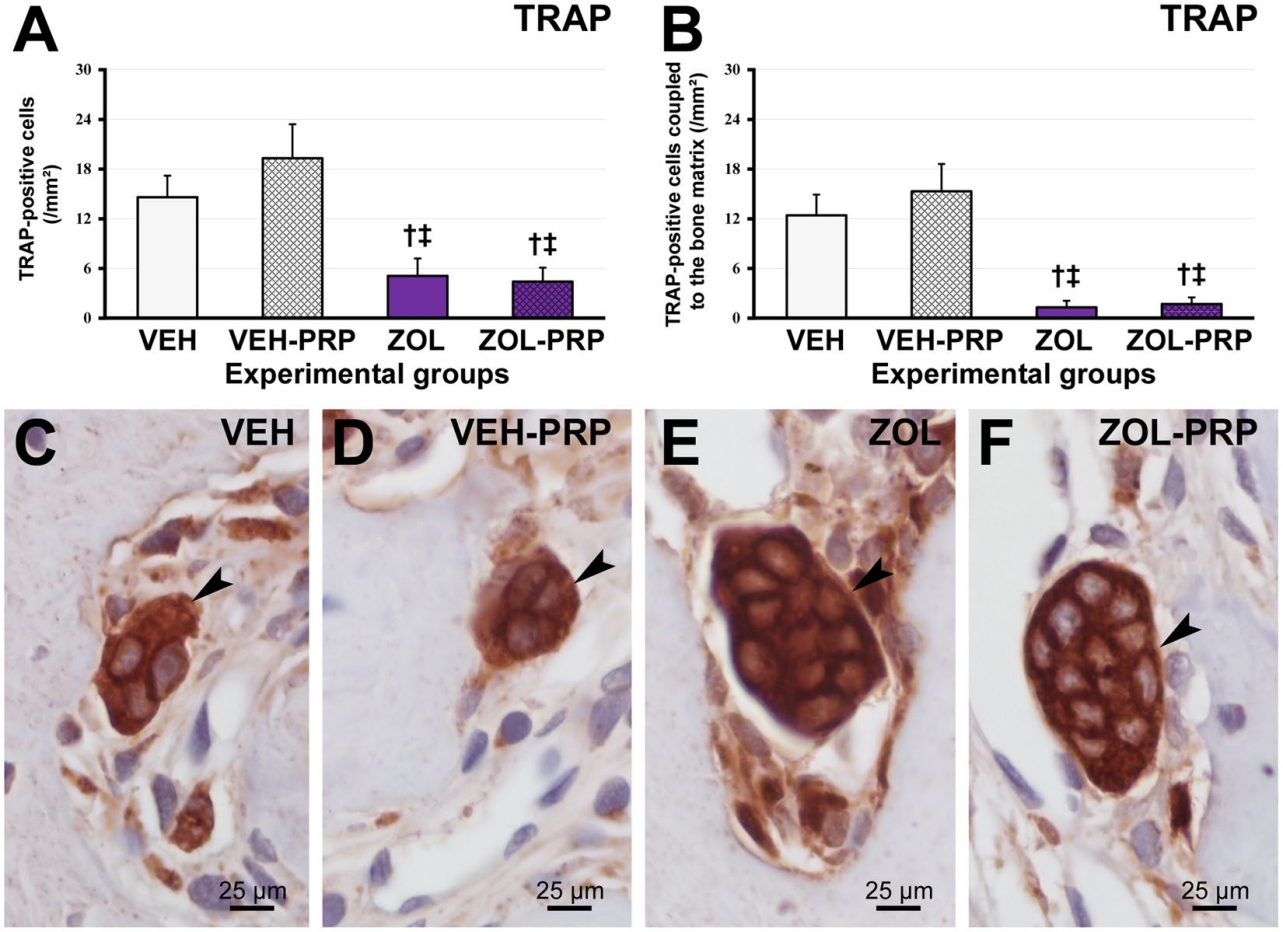
Immunolabeling pattern for TRAP in tooth extraction site. (**A–B**) Graphics presenting TRAP-positive cells (**A**) and TRAP-positive cells coupled to the bone matrix (**B**) in tooth extraction site in the different experimental groups at 28 postoperative days. (**C**–**F**) Photomicrographs showing immunolabeling pattern for TRAP in VEH (**C**), VEH-PRP (**D**), ZOL (**E**) and ZOL-PRP (**F**). Symbols: arrows, immunolabeling cells; ^†^Statistically significant difference in relation to VEH; ^‡^Statistically significant difference in relation to VEH-PRP; ^¶^Statistically significant difference in relation to ZOL. Original magnification: 1000×. Scale bars: 25 μm.

The immunohistochemical technique used for detection of TNFα, IL-1β, PCNA, VEGF, BMP2/4, OCN and TRAP showed specific immunolabeling for all proteins, which was confirmed by total lack of labeling in the negative control reactions. Immunolabeling showed a dark brown staining confined to the cytoplasm and to a lesser extent to the extracellular matrix for TNFα (Fig. 2), IL-1β (Fig. 2), VEGF (Fig. 3), BMP2/4 (Fig. 4), OCN (Fig. 4), confined exclusively to the nucleus for PCNA (Fig. 3) and confined exclusively to the cytoplasm for TRAP (Fig. 5).

### TNFα and IL-1β immunolabeling in mucosa overlying tooth extraction site

In group ZOL, TNFα and IL-1β immunolabeling density in the connective tissue of the mucosa overlying the tooth extraction site was significantly larger than in the other groups. In ZOL-PRP, the immunolabeling for such pro-inflammatory cytokines was significantly lower than in ZOL group and was significantly higher than in VEH and VEH-PRP groups. There was no statistically significant difference between VEH and VEH-PRP groups (Fig. 2).

### PCNA and VEGF immunolabeling in mucosa overlying tooth extraction site

The number of PCNA-positive and VEGF-positive cells in the connective tissue of the mucosa overlying the tooth extraction site was significantly lower in ZOL than in VEH, VEH-PRP and ZOL-PRP groups. There was no statistically significant difference between VEH and ZOL-PRP groups. PCNA and VEGF immunolabeling was significantly higher in VEH-PRP than in ZOL-PRP group and did not differ statistically from VEH (Fig. 3).

### BMP2/4 and OCN immunolabeling in tooth extraction site

The amount of BMP2/4-positive and OCN-positive cells in ZOL was lower than in VEH, VEH-PRP and ZOL-PRP groups. BMP2/4-positive and OCN-positive cells were lower in ZOL-PRP than in VEH-PRP group. Finally, BMP2/4 and OCN immunolabelings were greater in VEH-PRP and smaller in ZOL-PRP when compared with VEH (Fig. 4).

### TRAP immunolabeling in tooth extraction site

The amount of TRAP-positive cells and TRAP-positive cells coupled to the bone matrix did not differ in ZOL and ZOL-PRP groups. However, these parameters were lower in ZOL and ZOL-PRP when compared to VEH and VEH-PRP groups (Fig. 5).

## Discussion

The use of antiresorptive drugs has increased significantly due to the aging of the population worldwide, as they constitute an effective therapeutic alternative for treatment of common diseases in the elderly^3–5^. Thus, patients making chronic use of antiresorptive drugs have also increased progressively in the dental office, frequently requiring dental interventions potentially capable of triggering MRONJ^34^. Hence, it is necessary to establish effective and safe preventive protocols during dental treatment. In this context, the purpose of this study was to evaluate the effectiveness of local application of autologous PRP in the tooth extraction site of rats exhibiting the major risk factors for MRONJ. The most important finding of this study was that local use of autologous PRP minimized the negative consequences of zoledronate on the wound healing process by stimulating tissue repair. Thus, PRP use is a favorable therapy for preventing the occurrence of MRONJ after tooth extraction.

Experimental models have contributed to the proposition and/or evaluation of preventive and/or curative therapies for MRONJ, constituting an important guideline for clinical research. In the present study, our research group designed an experimental model based on epidemiological studies. This experimental model displayed the main risk factors for MRONJ. Concerning the individual risk factor, female senile rats (20 months) were used, as the disease most frequently affects women at an advanced age^8,9^. Regarding the drug related risk factor, an oncologic dose of zoledronate was used, since this drug is the most potent BP linked to most cases of MRONJ^8,9^. As to local risk factors, experimental periodontitis was induced in the lower first molar, followed by extraction, considering the fact that tooth extraction with periodontal and/or periapical involvement is identified as an important precipitating factor^34,35^. In this experimental model, ZOL group showed a MRONJ condition, supported by clinical and histopathological analysis.

A recent systematic review and meta-analysis of clinical studies reported that autologous PRP application in the tooth extraction site improves repair of both soft and hard tissues^36^, corroborating the findings of the present study. Keratinocytes, fibroblasts and osteoblasts are the main cells that in an orchestrated way participate in the wound healing process and are among the main targets of the PRP biostimulatory action^23–28^. In contrast, studies have demonstrated that zoledronate treatment exerts negative effects on keratinocytes^37,38^, fibroblasts^39,40^ and osteoblasts^41,42^. The zoledronate action on such cells has great importance in both impaired tissue repair of the tooth extraction site and occurrence of MRONJ^11–14^, which is consistent with the results obtained in this study. Our data showed that autologous PRP application in the tooth extraction site was able to prevent the negative effects of zoledronate on such cells and tissues. In addition, epithelial tissue recovery, connective tissue repair, local inflammatory response modulation, alveolar bone neoformation and lower impairment of pre-existing bone tissue were observed in animals treated with zoledronate and autologous PRP.

Another undesirable aspect during zoledronate therapy is its action on the vasculature and main local angiogenesis modulators, which can be responsible for compromising the repair process of soft and hard tissues of the tooth extraction site. Thus, triggering avascular necrosis of alveolar bone tissue. Studies have shown that zoledronate adversely affects viability^43,44^, proliferation^45^, migration^44,45^, differentiation^46^ and endothelial cell tube formation^45^, besides increasing apoptosis rate in such cells^43,44^. Zoledronate also reduces migration ability and differentiation of endothelial progenitor cells^44,46^. Santini *et al*.^47^ showed a decrease in the amount of endothelial cells and endothelial precursor cells in peripheral blood of patients treated with zoledronate in oncologic doses. Other studies showed a severe decrease in angiogenesis of the oral mucosa of patients with MRONJ^48^ and reduction in the expression of its major modulation factors, such as VEGF^49^, similar to the data from the present study. One of the indications that tissue repair was also stimulated by a favorable performance of PRP on vascular elements was an increased VEGF immunolabeling noticed in group ZOL-PRP.

Nitrogenous BPs, such as zoledronate, have osteoclasts as their main target cell^1,2^. This drug acts by blocking the action of the farnesyl diphosphate synthase enzyme, which belongs to the mevalonate pathway^1,2^. Blockage of this pathway inhibits osteoclastogenesis, prevents the activation of mature osteoclasts and induces premature apoptosis inactive osteoclasts^1,2^. A significant reduction in osteoclasts and osteoclasts coupled to the bone matrix in the tooth extraction site and surrounding areas was observed in ZOL and ZOL-PRP groups, confirming the effectiveness of the zoledronate treatment. Although some *in vitro* studies show that PRP promotes reduction in osteoclastogenesis^50,51^, the present study did not observe changes in the number of osteoclasts caused by the use of PRP, presumably due to the potent effect of zoledronate on osteoclasts and their precursors.

Another action of PRP, which probably contributed to the results observed in this study, was its anti-inflammatory action^31^. ZOL showed greater local inflammatory response and higher levels of key proinflammatory cytokines (TNFα and IL-1β) in the tooth extraction site. These data are in agreement with Morita *et al*.^52^, who suggested a close relationship between high levels of the proinflammatory cytokines TNFα, IL-1 and IL-6 in MRONJ pathogenesis, since mice deficient for these cytokines were resistant to developing osteonecrosis. In contrast, it was found that PRP use stimulated modulation of local inflammatory response in animals treated with zoledronate in our study. This was supposedly one of the responsible actions for promoting tissue repair process and preventing the occurrence of MRONJ after tooth extraction.

An additional systematic review including both *in vitro* and *in vivo* animal studies reported that platelet concentrates exhibit local antimicrobial action^32^. The present study was not aimed to perform an accurate microbiological evaluation. However, the histopathological analysis of the tooth extraction site from animals treated with zoledronate showed large areas of necrotic bone surrounded by bacterial colonies, corroborating previous studies^53–55^. Some studies demonstrated a close relationship between bacteria, especially of the Actinomyces genus, and MRONJ^56,57^; nevertheless, it is unclear whether it is a primary or secondary event. In group ZOL-PRP, the histopathological analysis of the tooth extraction site revealed no bacterial colonies attached to the bone tissue similar to those detected in group ZOL. Although the antimicrobial PRP action mechanism is not yet clear, some studies have shown an extensive bacteriostatic and bactericidal spectrum of action, including against many common microorganisms in the oral microbiota^58^. It should be taken into account that an antimicrobial action is among the benefits of PRP use. However, further microbiological studies are needed to elucidate this assumption.

Barba-Recreo *et al*.^59^ reported that rats treated with zoledronate and submitted to tooth extraction showed no improvement in the alveolar repair process after local allogeneic PRP application in the extraction site associated with mucoperiosteal flap. The use of allogeneic PRP may have compromised the benefits of this type of therapy. Such data and findings of this study highlight the importance of the origin of the PRP. In addition, the discrepancies between the scientific findings related to the use of PPR may be associated with the different preparation protocols^60^.

The present study evaluated the local use of autologous PRP as a preventative therapy for MRONJ due to the scarcity of studies on this topic. However, the combination of surgical lesion debridement and use of autologous PRP as a curative therapy for MRONJ have been investigated in experimental studies in animals and clinical studies. Sarkarat *et al*.^61^, performed curettage and local treatment with autologous PRP in a study using an experimental rat model after tooth extraction and clinical confirmation of the occurrence of MRONJ. These authors found no differences from the control group concerning epithelial regeneration, neoangiogenesis and bone sequestra formation; however, the amount of vital bone tissue at the surgical site was higher in rats treated with autologous PRP. Longo *et al*.^62^ and Coviello *et al*.^63^ comparatively evaluated the efficacy of isolated surgical therapy and surgical therapy associated with the local use of autologous PRP in clinical studies. Both studies found that the latter type therapy proved to be far more effective in treating MRONJ, which corroborates our findings and suggests that local use of autologous PRP can be a preventive and curative therapeutic option.

The occurrence of MRONJ compromises the quality of life of patients^64^. Treatment of this condition can be extensive, ineffective, may impair the treatment of the underlying disease and result in serious sequelae^17–19^. The use of preventive therapeutic strategies is ideal in the case of MRONJ. Considering the beneficial effects of PRP, such as biomodulatory action, local antimicrobial activity and absence of adverse effects, local employment of autologous PRP may constitute a promising preventive therapy for MRONJ. In addition, despite using an experimental animal model, the findings of this study may be important in guiding future research. Thus, human clinical studies are necessary for the establishment of preventive protocols to be used in patients making use of antiresorptive drugs and requiring invasive dental interventions.

## Conclusion

Within the limits of this study, it was concluded that local application of autologous PRP showed to be a viable, safe and effective preventative therapy to restore tissue repair capacity of the tooth extraction site that has been severely compromised by zoledronate treatment. The use of PRP showed a promising therapy to prevent the occurrence of MRONJ after tooth extraction.

## Methods

### Animals

The present study used twenty-eight senile (20 months) female rats (Wistar - *Rattus norvegicus*) with body weight ranging between 320–370 g. The animals were supplied by the School of Dentistry of Aracatuba (São Paulo State University – UNESP) and maintained throughout the experimental period under the following conditions: 12/12 light dark cycle at 22 ± 2 °C, relative humidity of 55 ± 5% and ventilation/exhaustion of 20 cycles per hour. The animals were maintained in plastic cages (41 × 34 × 18 cm) with three animals per cage, permitting free access to water and food. All procedures to avoid animal stress and to reduce the number of animals used were followed. The experimental protocol followed the rules established by the *“Guide for the Care and Use of Laboratory Animals”* ^65^ and was approved by the Ethics Committee on Animal Use at the School of Dentistry of Araçatuba (00581-2013). This research is in accordance with ARRIVE (Animal Research: Reporting of *In Vivo* Experiments)^66^.

### Anesthesia

Surgical procedures (ligation installation, cardiac puncture, tooth extraction and euthanasia) were performed under general anesthesia with ketamine (80 mg/Kg, Francotar®, Virbac, SP, Brazil) and xylazine (10 mg/Kg, Rompum®, Bayer, RS, Brazil).

### Ligature-induced periodontitis

**Figure 6:**
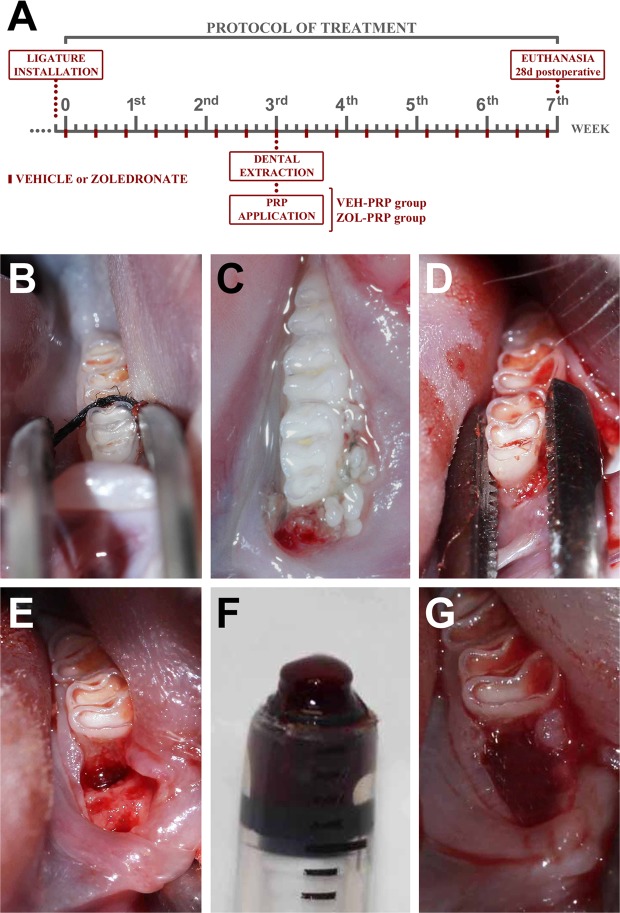
Experimental design. (**A**) Scheme illustrating experimental procedures performed during the study. (**B**) Ligature installed in lower first molar to induce experimental periodontitis. (**C**) Experimentally-induced periodontitis three weeks after ligature installation. (**D**) Surgical procedure for tooth extraction. (**E**) Clinical aspect of extraction site before PRP application. (**F**) Aspect of PRP activated. (**G**) Clinical aspect of extraction site after PRP application.

A cotton ligature (cotton thread #24, Linhas Corrente, SP, Brazil) was installed around the left first molar one day prior to the beginning of drug treatment (Fig. 6A,B). The ligature was maintained for three weeks in order to induce experimental periodontitis (EP) (Fig. 6C)^67,68^.

### Drug treatment plan

The drug treatment was initiated one day after ligature installation and lasted seven weeks (three weeks prior to exodontia and four weeks after exodontia) (Fig. 6A). Vehicle or zoledronate was administered (Sigma Chemical, St. Louis, MO, USA) intraperitoneally every three days. The vehicle consisted of 0.45 mL of 0.9% sodium chloride solution. The zoledronate dose was 100 μg/Kg, diluted in 0.45 mL of vehicle^67,68^. The drug treatment plan and zoledronate dose followed a protocol used for complementation of human cancer therapy adapted to the rat^69^.

### Experimental groups

Three weeks after beginning the drug treatment, the rats were randomly distributed in four groups: VEH, VEH-PRP, ZOL and ZOL-PRP. The following drug and local procedures were performed: VEH (n = 7), treated with vehicle and submitted to tooth extraction and no local treatment; VEH-PRP (n = 7), treated with vehicle and submitted to tooth extraction and application of autologous PRP on extraction site; ZOL (n = 7), treated with zoledronate and submitted to tooth extraction and no local treatment; ZOL-PRP (n = 7), treated with zoledronate and submitted to tooth extraction and application of autologous PRP on extraction site (Fig. 6A).

### PRP preparation protocol

Soon after the allocation of the animals in the different experimental groups, 1.5 ml of blood was collected via cardiac puncture in a syringe containing 0.15 mL of 3.2% sodium citrate. In groups VEH and ZOL the collected blood was discarded. In groups VEH-PRP and ZOL-PRP the collected blood was used to prepare the PRP. A sample of 25 µl of this blood from each animal in groups VEH-PRP and ZOL-PRP was also used in a blood smear for platelet count. The PRP preparation followed an adaptation of the protocol of Sonnleitner *et al*.^70^. Briefly, the blood samples were centrifuged at 160 G during 20 minutes for separation of blood plasma containing platelets, which was removed and centrifuged again at 400 G for 15 minutes. The platelet-poor plasma was discarded and 125 μl of PRP was separated for further use (100 µl was used to prepare the PRP gel and 25 µl reserved to be used in a PRP smear for qualitative and quantitative platelet evaluation. All centrifugation steps were performed in a refrigerated centrifuge (22 °C, Hermle Centrifuge Z323K, Hermle Labortechnik GmbH, Germany) and handling of samples was performed in a laminar flow hood (Flow Cab Vertical Laminar Veco®, Veco of Brazil Industry and Commerce Ltd. Equipment, Brazil).

### Tooth extraction

Tooth extraction was performed soon after the blood collection for PRP preparation. Briefly, the ligature was removed, then antisepsis of the oral cavity, sindesmotomy, dislocation (Fig. 6D) and extraction of the first lower left molar (Fig. 6E) was done using adapted dental instruments^67,68^. No local treatment was performed in groups VEH and ZOL, that is, immediately after extraction, the edges of the surgical wounds were sutured with 4-0 silk thread (Johnson and Johnson), in order to assist the wound healing process.

### Autologous PRP application in the tooth extraction site

Autologous PRP was used in the tooth extraction site in VEH-PRP and ZOL-PRP groups. The volume of PRP was submitted to activation with 10% calcium chloride (ScienceLab, TX, USA) (Fig. 6F) and placed in the center of the extraction socket (Fig. 6G), with the aid of a micropipette with an adapted tip. Next, the edges of the surgical wounds were sutured, as previously described, in order to assist the wound healing process and to guarantee the containment of the PRP gel activated inside the socket.

### Euthanasia and sample collection

Euthanasia was carried out seven weeks after the beginning of the drug treatment, that is, 28 days after tooth extraction. The animals were deeply anesthetized and transcardially perfused with 0.9% sodium chloride added with 0.1% heparin (100 ml), followed by fixative solution (800 ml) of 4% formaldehyde (Sigma, Saint Louis, MO, USA) in phosphate buffered saline (PBS - Sigma, St Louis, MO, USA), 0.1 M, 4 °C, pH 7.4. The hemimandibles were carefully dissected and submitted to post-fixation in the same fixative solution for 72 hours.

### Qualitative and quantitative analysis of platelet

Samples of 25 μl blood and 25 μl of PRP obtained from animals in groups VEH-PRP and ZOL-PRP were used for cytological smear and stained with a Romanovsky-type mixture (Fast Panotic LB Laborclin, SP, Brazil). These samples were then submitted to quantitative and qualitative analysis of platelets in light microscopy.

### Histological processing

The hemimandibles were demineralized in 10% ethylenediamine tetraacetic acid (EDTA) (Chemical® Sigma) in PBS for 60 days and submitted to conventional histological processing for paraffin embedding. Histological sections (5 µm thickness) of the extraction socket portion formerly occupied by the mesial and distal roots of the left first molar were collected from lingual to vestibular in appropriate histological slides.

The histological sections were submitted to hematoxylin-eosin staining (HE) for histopathological analysis of the tooth extraction site and adjacent tissues, and for histometric analysis of newly formed bone tissue (NFBT) and non-vital bone tissue (NVTB). For immunohistochemical analysis, the histological sections were divided into seven batches and submitted to indirect immunoperoxidase technique. The immunohistochemical processing followed the protocol described previously by Ervolino *et al*.^68^. In summary, the histological sections were incubated for 24 hours with one of the following primary antibodies: goat anti-TNFα (1:100; SC-1348, Santa Cruz Biotechnology, Santa Cruz, CA, USA), goat anti-IL-1β (1:100; SC-1252, Santa Cruz Biotechnology), mouse anti-PCNA (1:200; VP-P980, Vector Laboratories Inc., Burlingame, CA, USA), mouse anti-VEGF (1:200; SC-7269, Santa Cruz Biotechnology), rabbit anti-BMP2/4 (1:150; SC-9003, Santa Cruz Biotechnology), goat anti-OCN (1:100; SC-18319, Santa Cruz Biotechnology) and goat anti-TRAP (1:100; SC-30833, Santa Cruz Biotechnology). For signal amplification, universal biotinylated secondary antibodies (for 1.5 hours) and streptavidin conjugated with horseradish peroxidase (HRP) (for 1.5 hours) (Universal Dako Labeled HRP Streptavidin-Biotin Kit^®^; Dako Laboratories, CA, EUA) were used. The reaction was developed using the chromogen 3,3′-diaminobenzidine tetrahydrochloride (DAB chromogen Kit^®^; Dako Laboratories). As negative control, the specimens were submitted to the same procedures, eliminating the use of the primary antibody.

## Analysis of Results

### Analysis of the general health condition and intra-oral clinical examination

The general health condition of the animals was observed throughout the experimental period and the body weight was monitored weekly. Intra-oral clinical examination was performed, consisting of a detailed visual inspection of the oral cavity, in particular, of the tooth extraction site. The evaluated clinical parameters and their respective scores were based on Statkievicz *et al*.^67^ and Ervolino *et al*.^68^ and are shown in Table 2. Such data were expressed as medians and interquartile ranges of attributed scores in each evaluated parameter.

### Microscopic analysis

Microscopic analyzes were performed by a certified histologist (EE) who was blinded to treatments. For histopathological and histometric analyzes, three histological sections from the buccal, middle and lingual portion of the extraction socket were used. A histological section of the central portion of the extraction socket was used for immunohistochemical analysis.

### Region of interest (ROI)

In the present study, ROI (I), ROI (II) and ROI (III) were considered according to the performed microscopic analysis.

ROI (I) comprised a panoramic view of the tooth extraction site and adjacent tissues, consisting of a 4 mm × 4 mm area of the portion of the extraction socket previously occupied by the mesial and distal roots of the lower left first molar and adjacent tissues. The distal limit consisted of a line situated parallel to the surface of the coronary and radicular dentin of the lower left second molar, extending 4 mm mesially. The coronary limit consisted of a line located parallel to the gingival-cement boundary of the lower left second molar, extending 4 mm apically^67,68^.

ROI (II) comprised samples of the tissue overlying the tooth extraction site, consisting of two 250 μm × 250 μm areas located in the tissue overlying the tooth extraction site. The delimitation of this area followed a line located at the center of the connective tissue, perpendicular to the long axis of the teeth and dividing such tissue at its coronal apical direction. Two other lines were used, one parallel to the central portion of the cavity formerly occupied by the mesial root and another one parallel to the central portion of the cavity formerly occupied by the distal root of the first molar. The intersection of these lines determined the center of the two analyzed areas^67,68^.

ROI (III) concomitantly encompassed samples of pre-existing bone tissue in the alveolar wall and newly formed bone tissue inside the extraction socket, both vital tissues. An area of 500 μm × 500 μm located in the apical portion of the extraction socket within the space previously occupied by the mesial root or the distal root was analyzed. The center of these areas was positioned at the boundary between the pre-existing bone tissue and the newly formed vital bone tissue inside the extraction socket, both vital tissues. Only specimens with the characteristics of ROI (III) were evaluated. Specimens that exclusively had non-vital bone tissue and/or total absence of newly formed bone tissue were not included in this analysis.

### Histopathological analysis of the extraction site and adjacent tissues

The following parameters were evaluated in ROI (I) under light microscopy: (1) intensity of local inflammatory response; (2) extension of inflammatory process; (3) cellular and structure pattern of epithelial tissue; (4) cellular and structure pattern of connective tissue; (5) cellular and structure pattern of bone tissue; (6) contamination pattern of tooth extraction site. The evaluated histological parameters and their respective scores were based on Statkievicz *et al*.^67^ and Ervolino *et al*.^68^ and are shown in Table 2. Such data were expressed as medians and interquartile ranges of attributed scores in each evaluated parameter.

### Histometrical Analysis of NFBT and NVBT

In ROI (I), images were captured by using a digital camera (AxioCam® Carl Zeiss, Gottingen, Germany) coupled to an optical microscope (AxioLab®) and connected to a microcomputer. With the aid of image analysis software (Axiovision 4.8.2® Carl Zeiss), the total amount of bone tissue was measured; then, the percentage of newly formed bone tissue (NFBT) and percentage of non-vital bone tissue (NVBT) were calculated^68^. The NVBT consisted of regions where more than ten adjacent osteocyte lacunae were empty or containing osteocyte necrotic debris. The percentage of NFBT and NVBT was expressed as mean ± standard deviation.

### Immunohistochemical analysis of TRAP in tooth extraction site

In ROI (I), images of histological sections immunolabeled with TRAP were obtained as previously described. With the aid of image analysis software (Axiovision 4.8.2® Carl Zeiss), TRAP-positive cells and TRAP-positive cells coupled to the bone matrix were quantified^68^. The amount of TRAP-positive cells per mm² of bone was expressed as mean ± standard deviation.

### Immunohistochemical analysis of TNFα and IL-1β in the mucosa overlying the tooth extraction site

In ROI (II), images of histological sections immunolabeled with TNFα and IL-1β were obtained as previously described. The area corresponding to the immunolabeling was obtained through immunolabeling density using a color threshold tool of image analysis software (Axiovision 4.8.2® Carl Zeiss)^67,68^. The immunolabeling density per area was expressed in percentage as mean ± standard deviation.

### Immunohistochemical analysis of PCNA and VEGF in the mucosa overlying the tooth extraction site

In ROI (II), images of histological sections immunolabeled with PCNA and VEGF were obtained as previously described. PCNA-positive cells and VEGF-positive cells were quantified with the aid of an image analysis software (Axiovision 4.8.2® Carl Zeiss). The amount of immunolabeled cells per mm² was expressed as mean ± standard deviation.

### Immunohistochemical analysis of BMP2/4 and OCN in bone tissue of tooth extraction site

In ROI (III), images and analysis of histological sections immunolabeled with BMP2/4 and OCN were obtained as described above. The amount of immunolabeled cells per mm² was expressed as mean ± standard deviation.

### Statistical analysis

The Bioestat 5.3 (https://www.mamiraua.org.br/pt-br/downloads/programas/bioestat-versao-53; Mamiruá Institute, Manaus, AM, Brazil) program was used. The sample size was calculated to ensure 95% statistical test power (p < 0.05). For clinical and histopathological analyses, nonparametric Kruskal-Wallis analysis of variance test and Student-Newman-Keuls post-test were used. Shapiro-Wilk test was used for analysis of the normal distribution of data. For histometric analysis and immunohistochemistry, analysis of variance (ANOVA) and Tukey’s post-test were used, considering p < 0.05 as statistically significant.

## Data Availability

Data from this manuscript are available from Scientific Reports.
